# The potential role of serum angiotensin-converting enzyme in coronavirus disease 2019

**DOI:** 10.1186/s12879-020-05619-x

**Published:** 2020-11-25

**Authors:** Zhe Zhu, Ting Cai, Lingyan Fan, Kehong Lou, Xin Hua, Zuoan Huang, Guosheng Gao

**Affiliations:** 1 Department of Blood Transfusion, HwaMei Hospital, University of Chinese Academy of Sciences, Ningbo, P.R. China; 2 Ningbo Institute of Life and Health Industry, University of Chinese Academy of Sciences, Ningbo, P.R. China; 3 Key Laboratory of Diagnosis and Treatment of Digestive System Tumors of Zhejiang Province, Ningbo, P.R. China; 4 Department of Acute Infectious Diseases, HwaMei Hospital, University of Chinese Academy of Sciences, Ningbo, P.R. China; 5 Department of Clinical Laboratory, HwaMei Hospital, University of Chinese Academy of Sciences, 41 Xibei street, Ningbo, P.R. China; 6 Department of Experimental Medical Science, HwaMei Hospital, University of Chinese Academy of Sciences, Ningbo, P.R. China

**Keywords:** COVID-19, SARS-CoV-2, Serum ACE, Clinical significance

## Abstract

**Background:**

To explore the clinical significance of serum angiotensin-converting enzyme (ACE) activity in coronavirus disease 2019 (COVID-19).

**Methods:**

In this retrospective study, a total of 136 consecutive patients with confirmed COVID-19 were recruited. Demographic and clinical data were recorded. The serum ACE activity was measured at baseline and during the recovery phase, and its relationship with clinical condition was analyzed.

**Results:**

Of the 136 patients with confirmed COVID-19, the 16 severe patients were older and had a higher body mass index (BMI) and proportion of hypertension than the 120 nonsevere patients. In comparison to those of normal controls, the baseline serum ACE activities of subjects in the severe group and nonsevere group were decreased, with the lowest level in the severe group. However, the serum ACE activity increased in the recovery phase, and there were no significant differences among the severe group, nonsevere group and normal control group.

**Conclusion:**

Serum ACE activity could be used as a marker to reflect the clinical condition of COVID-19 since low activity was associated with the severity of COVID-19 at baseline, and the activity increased with the remission of the disease.

## Background

The newly emerged severe acute respiratory syndrome coronavirus 2 (SARS-CoV-2) and associated coronavirus disease 2019 (COVID-19) have produced a tremendous global health burden since December 2019. COVID-19 is characterized by fever, dyspnea, dry cough, headache and pneumonia, ranging from mild symptoms to respiratory failure, acute respiratory distress syndrome (ARDS), heart failure, sepsis, and septic shock [1]. The World Health Organization (WHO) announced that it is a public health emergency of international concern (PHEIC) on January 30, 2020 (https://www.who.int/).

The renin–angiotensin system (RAS) is well known for its ability to maintain blood pressure and electrolyte balance. In addition, it has been implicated in the pathogenesis of ARDS [2]. RAS has two axes, classic RAS: the ACE/Angiotensin (Ang) II/Ang II type 1 (AT_1_) receptor axis; and nonclassic RAS: the ACE2/Ang 1–7/Mas receptor (MasR) axis. The former deteriorates the impaired respiratory conditions, while the latter plays a protective role in ARDS [2, 3]. Determining what role RAS plays in COVID-19 is helpful to manage and treat this disease. As the receptor of SARS-CoV-2, numerous reports have focused on ACE2 [4–6]. Recently, soluble human ACE2 has been proven in vitro to have a potential therapeutic role in treating patients infected with SARS-CoV-2 [4]. However, ACE, which counterbalances ACE2 in the RAS, is poorly understood in COVID-19.

ACE is localized on the luminal surface of the endothelium, with the highest activity in lung capillary endothelial cells, and is also present in the blood [7]. Beginning as early as the 1980s, serum ACE activity has been used as a biomarker to reflect the damage of endothelial cells and the severity of ARDS caused by other pathogens [7, 8]. Therefore, we performed a retrospective study to explore the clinical significance of serum ACE activity in COVID-19.

## Methods

### Data collection

From January 23 to March 28, 2020, 136 patients with confirmed COVID-19 and 60 age- and sex-matched normal controls were included in this retrospective, single-center study. This study was approved by the institutional ethics committee (PJ-NBEY-KY-2020-061-01), and written informed consent was obtained from each patient. The demographic and clinical data were recorded from the electronic medical record (EMR).

Positive SARS-CoV-2 nucleic acid by real-time reverse transcription polymerase chain reaction (RT-PCR) of throat swab, nasopharynx swab and sputum specimens was defined as a confirmed case. The severity of the disease was distinguished according to the Diagnosis and Treatment Guidance of Coronavirus Diseases 2019 (Tentative Sixth Edition), National Health Commission (NHC) of the People’s Republic of China. Patients in the severe group met at least one of the following criteria: 1. respiration rate (RR) more than 30 times per minute; 2. resting oxygen saturation less than 93%; 3. ratio of partial pressure of arterial oxygen (PaO_2_) to fraction of inspired oxygen (FiO_2_) less than 300 mmHg; and 4. lesions rapidly progressing by more than 50% within 24–48 h on pulmonary imaging. Otherwise, they were classified into the nonsevere group.

The baseline stage refers to the period within 5 days of admission in this study, while the recovery phase was defined as the stage when the samples tested negative for SARS-CoV-2 and symptoms improved obviously.

### Serum ACE activity determination

Serum ACE activity was determined according to the manufacturer’s instructions (Quark Biotechnology Co., Ltd., Zhejiang, China) and analyzed on an ADVIA 2400 Chemistry System (Siemens, Germany). It was measured by a kinetic spectrophotometric assay that uses 3-(2-furylacryloyl)-L-phenylalanyl-glycyl-glycine (FAPGG) as the substrate. Briefly, ACE hydrolyzes FAPGG into furylacryloyl-L-phenylalanine (FAP) and glycyl-glycine (GG), with a decrease in absorbance at 340 nm, and the decreasing rate is directly proportional to the ACE activity [9].

### Statistical analyses

Statistical analyses were performed with GraphPad PRISM 5.0 software (GraphPad Software, San Diego, CA, USA) and SPSS statistical 16.0 software (IBM, Armonk, NY, USA). Continuous data with a normal distribution were expressed as the mean ± standard deviation (SD), and statistical significance between two independent groups was determined by unpaired Student’s *t*-test. The continuous variables with skewed distribution were expressed as medians and interquartile ranges (IQRs). Statistical significance among multiple groups was tested by the Kruskal-Wallis test, followed by post hoc comparisons with the Nemenyi test. The Mann-Whitney U test was performed for nonparametric independent two-group comparisons. Proportions for categorical variables were compared by the chi square or Fisher’s exact test. A Spearman correlation analysis was used to calculate the correlation coefficients. The independent factors influencing serum ACE activity were determined by multiple linear regression. Finally, we performed a multivariate logistic analysis to identify independent risk factors for the severity of COVID-19.

## Results

### Demographic and clinical features

There were 136 consecutive hospitalized patients with confirmed COVID-19 enrolled in this study, of who 51 (37.50%) were male, and 85 (62.50%) were female. The mean age was 50.17 years (SD: 15.73). Eleven (8.09%) and 8 (5.88%) patients had a history of smoking and drinking, respectively. Hypertension (33 [24.26%]) was the most common coexisting disorder. Fever (86 [63.24%]), cough (62 [45.59%]), fatigue (27 [19.85%]) and expectoration (32 [23.53%]) were the most common initial symptoms. According to the severity of disease, these patients were divided into two groups: the severe group (*n* = 16) and the nonsevere group (*n* = 120). The severity of disease was related to older age (57.50 ± 11.70 years vs 49.19 ± 15.98 years, *P* = 0.047), higher body mass index (BMI) (26.04 ± 5.63 kg/m^2^ vs 23.60 ± 3.33 kg/m^2^, *P* = 0.018), and higher proportion of hypertension (8 [50%] vs 25 [20.83%], *P* = 0.011). Diastolic and systolic blood pressure showed no significant differences between the two groups at admission (Table 1).

**Table 1:** Demographic and clinical features of patients with confirmed COVID-19

Variables	All patients (*n* = 136)	Nonsevere group (*n* = 120)	Severe group (*n* = 16)	*P* Value
Gender (%)				0.582
Male	51 (37.50)	44 (36.67)	7 (43.75)	
Female	85 (62.50)	76 (63.33)	9 (56.25)	
Age (years)	50.17 ± 15.73	49.19 ± 15.98	57.50 ± 11.70	0.047
Body mass index (kg/m^2^)	23.89 ± 3.77	23.60 ± 3.33	26.04 ± 5.63	0.018
Systolic blood pressure (mmHg)	132.79 ± 18.25	132.33 ± 17.76	136.19 ± 21.91	0.429
Diastolic blood pressure (mmHg)	78.75 ± 11.13	78.29 ± 10.76	82.19 ± 13.53	0.190
Smoking history (%)	11 (8.09)	10 (8.33)	1 (6.25)	0.774
Drinking history (%)	8 (5.88)	6 (5.00)	2 (12.50)	0.231
Coexisting disorders (%)	56 (41.18)	44 (36.67)	12 (75.00)	0.003
Diabetes	10 (7.35)	10 (8.33)	0 (0.00)	0.490
Hypertension	33 (24.26)	25 (20.83)	8 (50.00)	0.011
Cardiovascular disease	6 (4.41)	4 (3.33)	2 (12.50)	0.303
Hepatic disease	7 (5.15)	5 (4.17)	2 (12.50)	0.415
Chronic lung disease	7 (5.15)	5 (4.17)	2 (12.50)	0.415
Cancer	5 (3.68)	4 (3.33)	1 (6.25)	> 0.999
Initial symptoms (%)				
Fever	86 (63.24)	73 (60.83)	13 (81.25)	0.112
Nasal congestion	6 (4.41)	6 (5.00)	0 (0)	0.79
Sore throat	18 (13.24)	16 (13.33)	2 (12.5)	> 0.999
Headache/ Dizziness	10 (7.35)	9 (7.50)	1 (6.25)	> 0.999
Chill	18 (13.24)	4 (3.33)	4 (25.00)	0.278
Dry mouth	1 (0.74)	0 (0.00)	1 (6.25)	0.234
Fatigue	27 (19.85)	24 (20.00)	3 (18.75)	> 0.999
Anorexia	3 (2.21)	2 (1.67)	1 (6.25)	0.790
Nausea	3 (2.21)	2 (1.67)	1 (6.25)	0.790
Myalgia	10 (7.35)	9 (7.50)	1 (6.25)	> 0.999
Chest distress	6 (4.41)	4 (3.33)	2 (12.50)	0.303
Cough	62 (45.59)	53 (44.17)	9 (56.25)	0.362
Expectoration	32 (23.53)	28 (23.33)	4 (25.00)	> 0.999
Diarrhea	5 (3.68)	5 (4.17)	0 (0.00)	0.901
Anosmia	3 (2.21)	3 (2.50)	0 (0.00)	> 0.999

Data are presented as mean ± standard deviation or n (%)

*P* values indicate the comparison between nonsevere group and severe group

*COVID-19* coronavirus disease 2019

### Clinical laboratory data

The baseline laboratory data of the nonsevere group, severe group, and normal control group are shown in Table 2. Fibrinogen, neutrophil%, neutrophil-to-lymphocyte ratio (NLR), sialic acid (SA), and C-reactive protein (CRP) were gradually increased in the normal control, nonsevere and severe groups. The platelet count, lymphocyte%, lymphocyte count and serum ACE activity gradually decreased in the normal control, nonsevere and severe groups. White blood cell (WBC) count and neutrophil count showed no significant differences among the three groups. The erythrocyte sedimentation rate was not tested in normal controls, and no significant difference was found between the nonsevere group and severe group subjects.

**Table 2:** The baseline laboratory parameters in the nonsevere group and severe group of COVID-19 and normal controls

Variables	Normal controls (*n* = 60)	Nonsevere group (*n* = 120)	Severe group (*n* = 16)	*P* value
Fibrinogen (mg/dl)	368.00 (290.70–401.00)	430.50 (361.50–549.25)^&^	574.15 (405.30–668.00)^&#^	< 0.001
Platelet count (× 10^9^/L)	223.00 (190.25–258.75)	206.50 (165.00–262.50)^&^	155.00 (125.75–206.00)^&#^	0.001
WBC count (×10^9^/L)	5.70 (4.90–6.70)	5.05 (4.20–6.80)	5.35 (4.13–7.53)	0.068
Neutrophil% (%)	57.95 (53.08–61.68)	66.50 (59.15–73.48)^&^	75.70 (64.53–88.98)^&#^	< 0.001
Lymphocyte% (%)	33.30 (28.65–38.98)	24.45 (18.93–32.08)^&^	17.45 (8.23–22.25)^&#^	< 0.001
NLR	1.65 (1.35–2.16)	2.74 (1.88–3.87)^&^	4.24 (3.00–10.87)^&#^	< 0.001
Neutrophil count (×10^9^/L)	3.28 (2.57–4.09)	3.30 (2.55–4.39)	3.89 (2.25–6.57)	0.539
Lymphocyte count (×10^9^/L)	1.88 (1.58–2.16)	1.23 (0.9–1.59)^&^	0.74 (0.47–1.18)^&#^	< 0.001
Sialic acid (mg/dl)	56.50 (52.85–62.08)	75.90 (67.8–85.95)^&^	85.75 (82.68–97.38)^&#^	< 0.001
C-reactive protein (mg/L)	0.67 (0.36–1.71)	7.71 (1.73–26.59)^&^	36.64 (15.33–69.94)^&#^	< 0.001
Erythrocyte sedimentation rate (mm/h) ^a^	/	66.00 (38.00–90.75)	89.00 (60.5–105.75)	0.071
Angiotensin-converting enzyme (U/L)	75.00 (57.75–98.50)	63.50 (51.50–75.75)^&^	49.00 (37.25–67.50)^&#^	< 0.001

Data are presented as medians and inter-quartile ranges

*P* values indicate the comparison among nonsevere group, severe group and normal controls

*COVID-19* coronavirus disease 2019, *WBC* white blood cell, *NLR* neutrophil-to-lymphocyte ratio

^*&*^*P* < 0.05 versus normal control, ^#^*P* < 0.05 versus nonsevere group

^a^The number of COVID-19 patients who tested erythrocyte sedimentation rate was 114 and 12 in the nonsevere and severe group, respectively, and it was not tested in normal controls

### Correlations between serum ACE activity and other variables

**Fig. 1:**
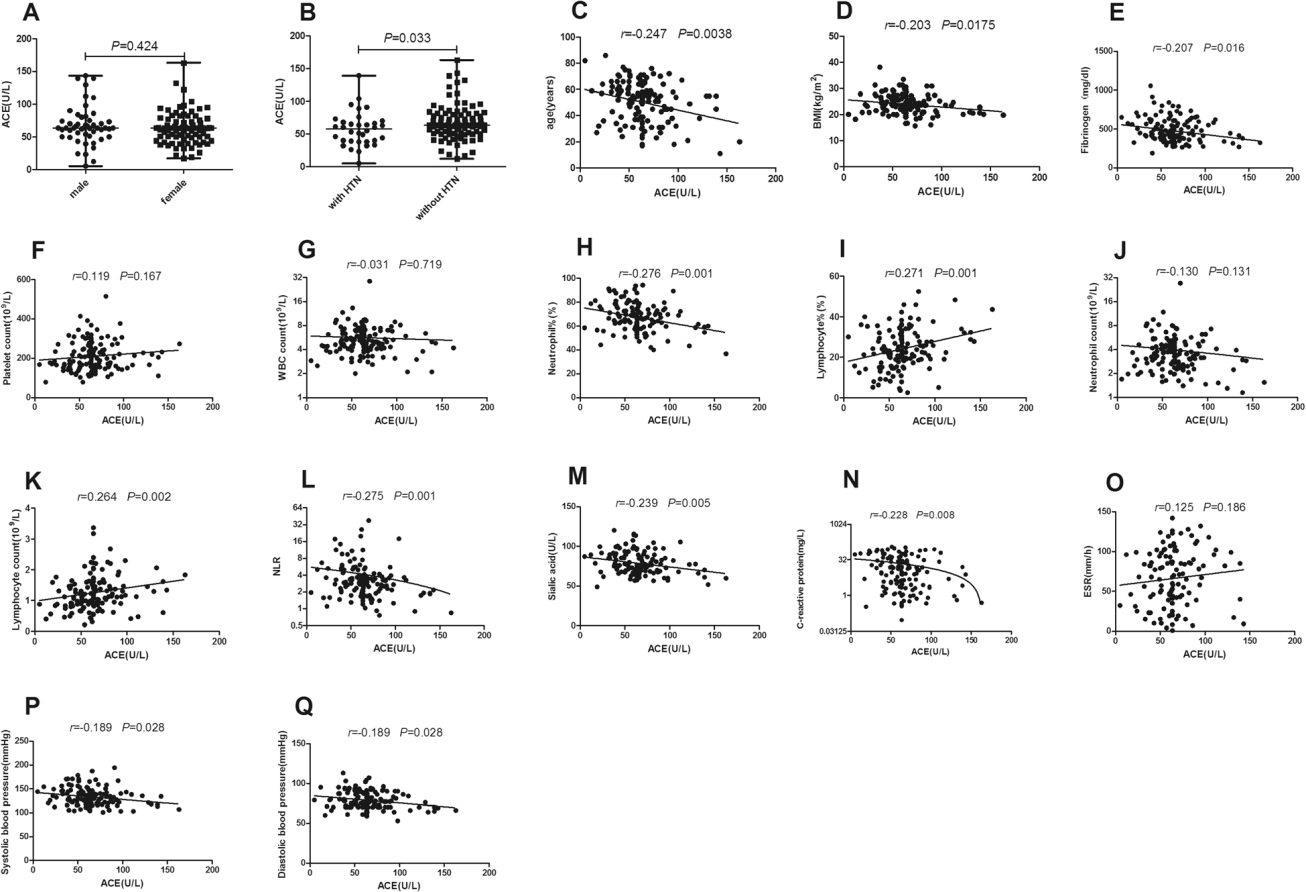
The association between baseline serum ACE activity and other variables in patients with COVID-19. The activity of serum ACE in male and female patients (**a**); the activity of serum ACE in patients with and without hypertension (**b**); correlations between serum ACE activity and age (**c**), BMI (**d**), fibrinogen (**e**), platelet count (**f**), WBC count (**g**), neutrophil% (**h**), lymphocyte% (**i**), neutrophil count (**j**), lymphocyte count (**k**), NLR (**l**), sialic acid (**m**), C-reactive protein (**n**), ESR (**o**), systolic blood pressure (**p**) and diastolic blood pressure (**q**). ACE: angiotensin-converting enzyme; COVID-19: coronavirus disease 2019; BMI: body mass index; WBC: white blood cell; NLR: neutrophil-to-lymphocyte ratio; ESR: erythrocyte sedimentation rate

The baseline serum ACE activity was negatively correlated with age (*r* = − 0.247, *P* = 0.0038), BMI (*r* = − 0.203, *P* = 0.0175), fibrinogen (*r* = − 0.207, *P* = 0.016), neutrophil% (*r* = − 0.276, *P* = 0.001), NLR (*r* = − 0.275, *P* = 0.001), SA (*r* = − 0.239, *P* = 0.005), CRP (*r* = − 0.228, *P* = 0.008), systolic blood pressure (*r* = − 0.189, *P* = 0.028) and diastolic blood pressure (*r* = − 0.189, *P* = 0.028) and positively correlated with lymphocyte% (*r* = 0.271, *P* = 0.001) and lymphocyte count (*r* = 0.264, *P* = 0.002), as revealed by Spearman correlation analysis (Fig. 1). Moreover, patients with hypertension had lower serum ACE activity than patients without hypertension. Serum ACE activity in the male and female patients was not significantly different. However, only neutrophil%, age and diastolic blood pressure had negative correlations with serum ACE activity, as revealed by multiple linear regression after adjusting for other confounders (Table 3).

**Table 3:** Multiple linear regression between serum ACE activity and other variables in patients with COVID-19

Variables	*B*	*S.E.*	*t* value	*P* value
Neutrophil% (%)	−0.519	0.185	−2.799	0.006
Age (years)	−0.302	0.139	−2.173	0.032
Diastolic blood pressure (mmHg)	−0.401	0.190	−2.113	0.036

*COVID-19* coronavirus disease 2019

### Dynamic changes in serum ACE activity

**Fig. 2:**
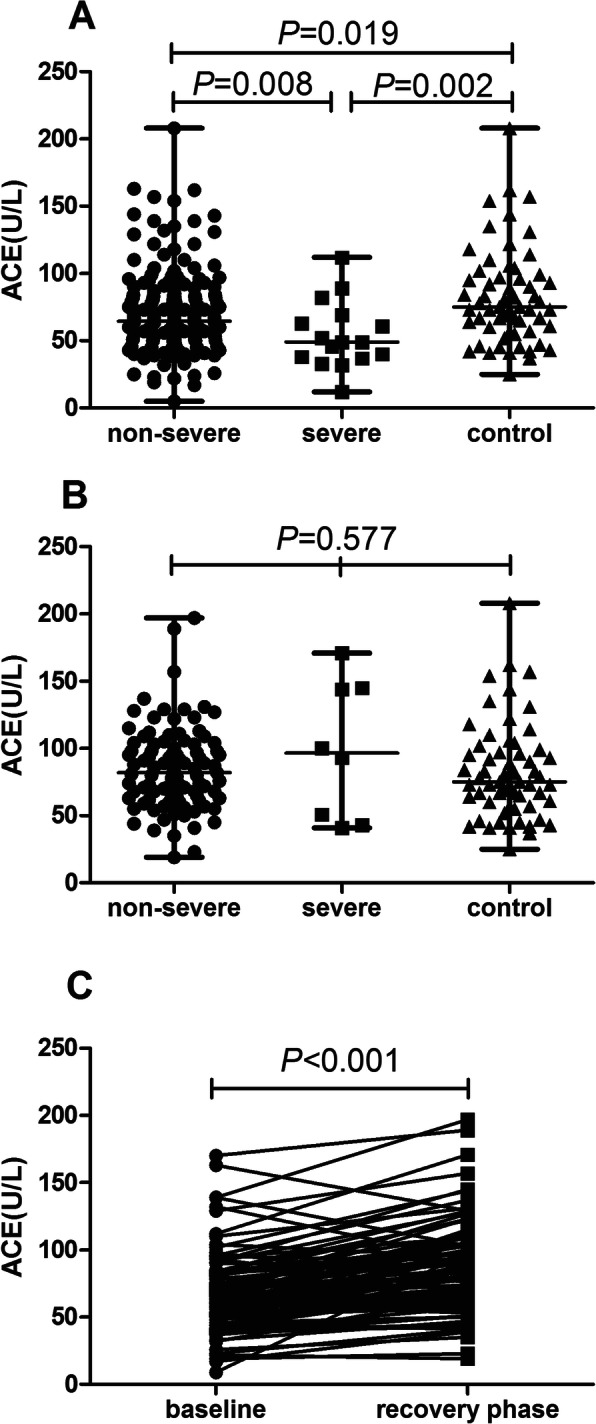
The comparisons of serum ACE activity among nonsevere group, severe group and normal controls at baseline (**a**) and recovery phase (**b**); the dynamic changes of serum ACE activity in patients with COVID-19 (**c**). Ninety-seven nonsevere cases and 8 severe cases tested serum ACE activity in the recovery phase

The baseline serum ACE activity in patients with COVID-19 was significantly lower than that in normal controls, with the lowest level in the severe COVID-19 patients. Among all included patients, 97 nonsevere and 8 severe subjects were tested for serum ACE activity in the recovery phase. It was demonstrated that the serum ACE activity in patients with COVID-19 increased gradually after treatment, with a significant difference from the baseline level (*P* < 0.001). Moreover, the serum ACE activity in the recovery phase in the severe and nonsevere groups showed no significant differences when compared with that in normal control group (Fig. 2).

### Logistic analysis

We performed a multivariate logistic analysis and found that BMI, hypertension and CRP were dependent risk factors for the severity of COVID-19, while serum ACE activity was not (Table 4).

**Table 4:** Logistic regression analysis of variables associated with the severity of COVID-19

Variables	Univariate analysis			Multivariate analysis		
	OR	95% CI	*P* value	OR	95% CI	*P* value
Age (years)	1.039	1.000–1.079	0.052			
Body mass index	1.186	1.032–1.363	0.016	1.175	1.011–1.366	0.035
Hypertension	4.886	1.657–14.409	0.004	4.393	1.389–13.895	0.012
Fibrinogen (mg/dl)	1.004	1.001–1.007	0.007			
Platelet count (×10^9^/L)	0.989	0.980–0.999	0.031			
Neutrophil% (%)	1.065	1.014–1.118	0.011			
Lymphocyte% (%)	0.928	0.874–0.986	0.015			
NLR	1.095	1.007–1.191	0.034			
Lymphocyte count (×10^9^/L)	0.160	0.041–0.616	0.008			
Sialic acid (mg/dl)	1.051	1.013–1.090	0.008			
C-reactive protein (mg/L)	1.024	1.008–1.041	0.004	1.022	1.004–1.040	0.016
Angiotensin converting enzyme (U/L)	0.978	0.954–1.002	0.074			

*COVID-19* coronavirus disease 2019, *NLR* neutrophil-to-lymphocyte ratio

## Discussion

In this retrospective study, we found that few patients (11.76%) with COVID-19 were severe. The severe patients were older, had a higher BMI and proportion of hypertension, and exhibited a tremendous change in peripheral immune-inflammatory parameters. It was demonstrated that fibrinogen, neutrophil%, NLR, SA, and CRP were increased and that platelet count, lymphocyte% and lymphocyte count were decreased in the severe cases when compared with those of the nonsevere cases and normal controls. This result was similar to our previous report [10], although we enlarged the sample size in this study. We also found that the baseline serum ACE activity in the severe group and nonsevere group were both decreased, with the lowest level in the severe group. Moreover, serum ACE activity at baseline was significantly correlated with most of the immune-inflammatory parameters, and it increased with the remission of the disease. Additionally, no significant difference was found among the severe group, nonsevere group and normal control group in the recovery phase. However, serum ACE activity could not serve as an independent risk factor for the severity of COVID-19.

Previous studies have revealed that serum ACE activity can be a marker to monitor the condition of ARDS. ARDS is the most severe form of acute lung injury (ALI) [11], which could be caused by various pathogenic conditions, such as influenza, SARS-CoV and SARS-CoV-2 infections [1, 12, 13]. The pulmonary endothelium plays a key role in the pathogenesis of ARDS, which is manifested by pulmonary endothelial cell damage and increased capillary permeability. ACE is distributed mainly along the luminal pulmonary endothelial surface, and its activity is affected by the function of the pulmonary endothelium. Direct evidence has shown that decreased pulmonary capillary endothelium-bound (PCEB)-ACE activity is correlated with the severity of lung disease in ARDS patients [14]. On the other hand, serum ACE originates from the capillary endothelium; therefore, pulmonary endothelium dysfunction indirectly influences ACE activity in the serum. Previous findings indicated that decreased serum ACE activity was closely correlated with the severity of ARDS and increased in the recovery phase [7, 8]. In line with previous studies, we speculated that the decreased serum ACE activity in severe COVID-19 was attributed to the injured pulmonary endothelium. Moreover, circulating ACE inhibitors (ACEIs) and proteolytic enzymes generated in critical illness may also play a role [7, 15]. In the recovery phase, the pulmonary capillary endothelium status improved, and ACEIs levels declined, which was reflected in the increased activity of ACE.

The imbalance in the ACE/AII/AT1R axis and the ACE2/Ang1–7/MasR axis is known to be involved in the pathogenesis of ARDS [2, 3]. Moreover, Glowacka et al. [16] found that the recombinant SARS-CoV spike protein bound to ACE2, downregulated ACE2 expression and promoted lung injury. Similar to SARS-CoV, SARS-CoV-2 utilizes ACE2 to enter human cells. It has also been reported that an increased level of plasma Ang II was strongly associated with lung injury severity in COVID-19 patients [17], which implied the activation of the ACE/AII/AT1R axis. Therefore, it is believed that ACE1/ACE2 imbalance occurs in COVID-19 [18, 19]. However, the ACE protein expression level in lung tissue is controversial. In a previous study published in Nature in 2005, Imai et al. [20] observed that ACE2 protein in the lung tissue of ARDS model mice was downregulated, while ACE levels remained constant. In another study, high ACE protein expression was found in the lung tissue of an ARDS animal model [21]. Although revealed by us and others [7, 8], serum ACE activity was decreased, it may not reflect ACE protein in the lung. Therefore, further studies are needed to explore the status of ACE protein in COVID-19, as well as its relationship with serum ACE activity.

The immune-inflammatory reaction plays a key role in the progression of COVID-19 [10, 22]. Therefore, we performed correlation analyses and found that serum ACE activity was significantly correlated with most of the immune-inflammatory parameters in this study. Additionally, we performed multiple linear regression to find that only neutrophil%, age and diastolic blood pressure had negative correlations with serum ACE activity after adjusting for other confounders. In agreement, severe patients were older and hypertensive, which further supports the role of serum ACE activity in the development of COVID-19.

Serum ACE activity is always increased in patients with hypertension, as revealed by previous studies [23, 24], which was different from our results. As far as we are concerned, hypertension resulted in worse deterioration of lung tissue, thus offsetting the originally increased serum ACE activity. On the other hand, ACEIs affect serum ACE activity. However, only one patient with hypertension was treated with an ACEI in our study, which would have little bearing on the overall result.

Serum ACE activity was detected by a kinetic spectrophotometric assay in this study. A kinetic spectrophotometric assay using FAPGG as a substrate was first introduced by Holmquist et al. in 1979 [25] and soon widely used on automated biochemical analyzers [26]. Similarly, we used the ADVIA 2400 Chemistry System, an advanced high-speed automated clinical chemistry analyzer, to test ACE activity.

Interestingly, some studies have reported that the ACE gene insertion/deletion (I/D) polymorphism of a fragment of 287 pairs of bases in intron 16 is associated with the incidence and consequence of ARDS in patients [27, 28], as well as related to the hypoxemia of SARS cases revealed by Itoyama et al. in Vietnamese individuals [29]. However, another study performed in Hong Kong did not discover a relationship between the ACE I/D polymorphism and the susceptibility to SARS-CoV infection or the outcome of SARS-CoV-infected patients [13]. A very recent report has indicated that the ACE I/D polymorphism may affect the spread and outcome of COVID-19 [30]. Additionally, an earlier study found that circulating ACE activity is influenced by the ACE I/D polymorphism [31]. Therefore, the relationship between the ACE I/D polymorphism, serum ACE activity and the outcome of patients with COVID-19 needs to be explored in the future.

To our own knowledge, this is the first study to investigate the role of serum ACE activity in the progression of COVID-19. However, this study has some limitations. First, ACE activity in bronchoalveolar lavage fluid and ACE protein expression in lung tissue, along with ACE2, angiotensin II, etc. was not tested. To better understand the role of RAS in the pathogenesis of COVID-19, the above markers should be investigated. Second, serum ACE activity could not serve as an independent risk factor for the severity of COVID-19 in this study. Third, some patients did not have their serum ACE activity tested at the recovery phase. Fourth, this was a single-center retrospective study with a relatively small sample size. Fifth, the unequal sample size in the severe and nonsevere groups might lead to biased results. These limitations must be considered when using this marker, and a multicenter, prospective cohort study with a larger sample size is needed to validate the role of serum ACE activity in COVID-19.

## Conclusion

Low activity of serum ACE at baseline was associated with the severity of COVID-19, and it increased with the remission of the disease. Therefore, serum ACE activity could be used as a marker to reflect the clinical condition of COVID-19. Using this marker may help us to manage the disease more efficiently.

## Data Availability

The datasets used and analyzed during the current study are available from the corresponding author on reasonable request.

## Abbreviations

COVID-19: coronavirus disease 2019
SARS-CoV-2: Severe acute respiratory syndrome coronavirus 2
ACE: Angiotensin-converting enzyme
ACE2: Angiotensin-converting enzyme 2
BMI: Body mass index
WHO: World health organization
ARDS: Acute respiratory distress syndrome
RAS: Renin–angiotensin system
EMR: Electronic medical record
RT-PCR: Real-time reverse transcription polymerase chain reaction
RR: Respiration rate
PaO_2_: Partial pressure of arterial oxygen
FiO_2_: Fraction of inspired oxygen
FAPGG: 3-(2-furylacryloyl)-L-phenylalanyl-glycyl-glycine
FAP: Furylacryloyl-L-phenylalanine
GG: Glycyl-glycine
NLR: Neutrophil-to-lymphocyte ratio
SA: Sialic acid
CRP: C-reactive protein
